# Evaluation of self-deception: Factorial structure, reliability and validity of the SDQ-12 (self-deception questionnaire)

**DOI:** 10.1371/journal.pone.0210815

**Published:** 2019-01-30

**Authors:** Carlos Sirvent, Juan Herrero, María de la Villa Moral, Francisco Javier Rodríguez

**Affiliations:** 1 Institute of Drug Dependence “Spiral,” Oviedo, Principado de Asturias, Spain; 2 Department of Psychology, University of Oviedo, Oviedo, Principado de Asturias, Spain; University of Texas at Austin, UNITED STATES

## Abstract

We all need to resort to deception, either with ourselves (denial, self-deception, mystification) or with others (with modalities, such as impression management, social desirability), to a greater or lesser extent. Lies, in their broader meaning, are interpreted as something rather adaptive, useful, and necessary in our socioaffective world. In particular, self-deception is a highly interesting psychological concept in the clinical population, namely, in drug dependents, as it serves as a mechanism for maintaining addiction. The objective of this study was to create and explore the validity and psychometric properties of a short self-deception scale (SDQ-12), derived from the IAM-40 and emphasizing the manipulation and mystification dimensions. Participants in this study included a group of drug dependents (alcoholics and drug abusers) under treatment (n = 417) as well as a group of adults from the general population (n = 124) (total N = 541), selected using simple random sampling. Across the sample, 63% of individuals were male, with a mean age of 38.65 years (S.D. = 10.61). Empirical exploration of the SDQ-12 items using exploratory and confirmatory factor analysis revealed that the instrument has a clear structure matching the theoretically relevant proposed dimensions of mystification and manipulation. Internal consistency was verified (Cronbach’s alpha coefficient = .85), and confirmatory factor analysis revealed that the two-dimensional model provided an appropriate fit to the data. In addition, manipulation was greater in young male individuals, with significant differences found in mystification and manipulation between the general population and alcoholics and drug abusers. Our study supports the clinical and research importance of the SDQ-12 scale, due not only to its diagnostic efficacy but also to its novel nature, its importance, and its relevance. It could be particularly useful for evaluating the substantial components of self-deception in the addict population, thus guiding therapists in their diagnostic and interventional role.

## Introduction

Self-deception as a process is a controversial concept and an object of debate among scholars. The Collins Dictionary defines self-deception as the act of deceiving oneself, especially about the true nature of one’s feelings or motivations. Basically, self-deception consists of a psychological process that originates and feeds a belief that is opposed to the evidence that the subject possesses. In this work, we will approach self-deception from a psychopathological perspective.

Self-deception is not in itself pathological. We all are capable of it and use it to a greater or lesser extent to interrelate. Pathological self-deception is based on a set of false beliefs, with harmful consequences for the subject’s mental health. Through a process of false self-conviction, subjects begin to fool themselves repeatedly until they come to believe their own deception through an implicit non-associative learning process [1]. Elements such as confirmation bias and cognitive dissonance feed back into this process.

Self-deception or unconscious deception has enormous clinical potential. Researchers have focused on apparently similar and dissimilar constructs, such as deception, lying, desirability and impression management (a process whereby someone tries to influence the observations and opinions of others about something). Even certain defense mechanisms, such as projection, rationalization and denial, are satellite concepts that surround the world of self-deception and mystification [2].

Self-deception affects people with substance abuse problems, making them less able to cope with imminent threats to their health [3]. During the self-deception process, these people try to maintain conviction in their beliefs or give themselves a sense of control over their world [4], that is, over their addiction.

It has been shown that, in the clinical population, specifically among drug addicts and emotional dependents, a syndromic profile of self-deception is more pronounced than in the general population [2]. The literature on addiction points out that substance abusers show more self-deception than those who do not abuse [5], which is not surprising since the abuse of drugs and alcohol are disorders characterized by denial, dishonesty and self-deception [6]. As a result, therapeutic intervention has shifted the focus towards the recovery of honesty and the capacity for objectification [7] as substantial elements in the treatment for dependency [8].

Self-deception has a negative impact on addiction in that there is an inverse relationship between self-deception and the duration of abstinence (individuals with higher self-deception scores are more likely to experience shorter periods of abstinence). Consequently, the level of arbitrary beliefs related to addiction are also higher. In Martínez-González’s [9] research, the importance of self-deception as a maintenance mechanism for drug addiction was demonstrated: Drug addicts presented high levels of denial, selective amnesia, projection and fantasized thinking. These authors also observed a significant relationship between nuclear beliefs related to consumption, craving and the level of self-deception.

Due to the important role played by self-deception in the recovery and treatment of addicts, research is necessary to provide clinicians with valid and reliable instruments to evaluate it.

Most self-deception measures available in the literature are related to a conceptualization of social desirability [10, 16] in which a move away from reality [14] and manipulation play important roles [17] (see Table 1). Decades ago, Damarin & Messick [18] suggested that self-deception implies the defensive distortion of private self-image to be consistent with a global evaluative bias; this idea was operationalized by Sackeim & Gut [19] and served as the basis for the Balanced Inventory of Desirable Responses (BIDR) [14, 15], in which two separate dimensions of desirable response were distinguished: self-deception and impression management.

**Table 1:** Instruments measuring the various components of self-deception.

INSTRUMENT	COMPONENT OF SELF-DECEPTION
*Edwards social desirability scale (1957)* [10]	Social desirability
*Crowne-Marlowe social desirability scale (1964)* [11]	Social desirability
*Eysenck Personality Inventory (EPI) (1964)* [12]	Lie scale
*Occupational Personality Questionnaire (SHL*, *Saville & Holdsworth 1999)* [13]	Social desirability
*Balanced Inventory of Desirable Responding (BIDR*, *Paulhus)* [14, 15]	IM scale: impression management, social desirability SDE scale: self-deception increase scale
*Escala de self-deception (IAM-40) de Sirvent (2012)* [2]	General scale: self-deception Subscales: insincerity, manipulation, reiteration, mechanisms of denial, mystification, distorted reality

Another relevant aspect often highlighted in the literature might accompany the evaluation of manipulation on scales of self-deception: contact with reality. The available empirical evidence suggests that self-deception can protect people’s beliefs and desires from a contrary reality (see Von Hippel & Trivers [17] for a review of related studies) and that self-deceivers can be systematically imprecise in their perceptions of reality. An important characteristic of self-deception is its permanent tension with true perception, which could explain why self-deception is related to so-called contact with reality. In the same way, deviation from reality is a characteristic of the desirable social response [20] described by Paulhus [15], which also applies to the dimension of self-deception.

A loss of contact with reality relates to the concept of "clinical mystification." [21] Clinical mystification consists of a peculiar form of self-deception that affects the subject’s vital activity and development, generating a shield of distrust that hinders interpersonal communication with communicative opacity and the systematic use of denial mechanisms [22]. Additionally, there are perceptual biases that in extreme but not infrequent cases lead to "deception as a way of life," including the assumption of a false external appearance and even the adoption of a misanthropic attitude. This stage is reached through an implicit non-associative learning process [22]. Mystification can occur in psychiatric pathologies, such as addiction, sociopathy or personality disorders.

The purpose of this study was to develop an instrument to evaluate pathological self-deception that includes its two essential dimensions: manipulation and mystification. As has been shown previously, the availability of pathological self-deception instruments is relatively scarce, and none of them is indicated as a screening instrument.

The existing tests (see Table 1) are limited to self-deception in general [2, 14, 15], desirability [11–15], impressions management [14, 15] (more typical of educational and occupational psychology) and the extent of lying [12], a scope that is not relevant here. Only the IAM-40 inventory explores related facets, but it does not have a scale for clinical mystification, and that test is more directed at clinical intervention than at screening [2], where the component of "clinical mystification" [21] is accentuated in addition to being an effective instrument for assessing level of self-deception in general. The present study explores the validity and psychometric properties of a brief 12-item self-deception scale, developed from the IAM-40 Inventory by Sirvent [2] and measuring the dimensions of manipulation and clinical mystification. It is assumed to be present in a large number of psychic processes, both normal and altered. Here, data were collected from subjects enrolled in a program for substance use disorders as well as adults from the general population.

## Materials and methods

### Participants

Participants in this study belonged either to a group of substance abusers receiving treatment in a recovery program (n = 417) or to a group of adults from the general population (n = 124) (total N = 541). Substance abusers were divided into two groups: alcoholics (n = 157) and drug abusers (n = 260). The distribution of gender and age across the groups was as follows: alcohol group (57% males, mean age = 43.47 years, S.D = 9.32); substance group (78% males, mean age = 35.07 years, S.D. = 8.48); and general population group (40% males, mean age = 40.03 years, S.D. = 13.17). In the complete sample, 63% were males, and the mean age was 38.65 years (S.D. = 10.61).

### Procedure and instruments

Informed consent was obtained from the participants in accordance with what is established in the "Ethical Principles for Human Research" in the Declaration of Helsinki. The research was reviewed by the Ethics Committee of the Department of Psychology at the University of Oviedo (Spain). Both the institutionalized addicts and the general population group agreed to participate in the study and signed the consent forms for participation. Because there were no participants under the age of 18, it was not necessary to request the consent of parents and guardians. All participants were informed that the data collected were for research purposes only.

#### Self-deception

A 12-item questionnaire of self-deception (SDQ-12, selected from Sirvent’s IAM-40 Inventory [2]) was administered to all participants in the study. Items from the original IAM-40 questionnaire were selected to measure the dimensions of clinical mystification and manipulation. Initially, 14 items were selected from the IAM-40 subscales to measure mystification. These 14 items measured biased reality (8 items), authenticated registry of reality (3 items), and denial mechanisms (3 items). The strategy was to include aspects closely related to clinical mystification that in larger scales are represented in differentiated subcategories but, in turn, involve very relevant aspects of clinical mystification. These 14 initial items were subsequently reduced to 6 after verifying that these 6 items adequately reflected the dimension of mystification (saturation greater than.50). For the scale of manipulation of reality, an analogous procedure was followed using the original manipulation subscale of the IAM-40 (8 items). Only those 6 items with the highest saturation (> .50) were retained.

As a result, the SDQ-12 measured two dimensions of self-deception: manipulation and mystification. Each subscale was ultimately composed of 6 items each, with responses adhering to a 5-point Likert scale (from 1 -strongly disagree- to 5 -strongly agree-). Mystification measures the respondent’s departure from reality in terms of not being aware of important aspects of their lives (*It takes me a while to become aware of certain key issues in my life*), inaccurate perceptions (*In important personal matters in my life*, *I think I repeatedly make the same mistakes*), and distortion about one’s lifestyle (*I sometimes feel my lifestyle is a sham*. *I live a lie*). Manipulation refers to the respondent’s self-presentation with the intention of influencing others’ behavior (*Honestly*, *I often choose to answer not with the truth but rather with whatever is most convenient for me*).

### Analyses

We explored the empirical structure of the 12 items of the SDQ-12 using both exploratory and confirmatory factor analysis techniques. Exploratory factor analysis was used as a preliminary procedure to ascertain whether the 12 items of the SDQ-12 equally clustered into theoretically meaningful factors. Internal consistency analyses for the complete questionnaire and for each factor were performed at this step. Then, we used confirmatory factor analysis for examination of the measurement model. We used EQS structural equation program. Maximum Likelihood estimation and Satorra-Bentler χ2 were used to correct departure from multinormality (as indicated by Mardia’s normalized estimate of multivariate kurtosis) for the calculation of robust fit indexes (CFI and RMSEA), standard errors, and statistical significance of the parameters.

Subsequently, with the purpose of establishing the predictive validity of the questionnaire for the various groups considered (general population, alcohol abusers, and drug abusers), multivariate analysis of variance (MANOVA) was used. In addition, ANOVA (one-factor analysis of variance) was used to analyze in-depth differences across groups in each of the subscales.

## Results and discussion

### Principal component and internal consistency analyses

Principal component analysis was carried out, and a solution of two components with eigenvalues greater than 1.0 was extracted and rotated obliquely (Promax-4). An inspection of the scree plot (not shown here for reasons of space) indicated that the point where the slope of the curve clearly levels off was in the step from the second (eigenvalue = 1.52) to the third component (eigenvalue = 0.84). This suggested that a two-factor solution was the most plausible. The Kaiser-Meyer-Olkin measure of sampling adequacy was.89, and the Bartlett test of sphericity was statistically significant (χ^2^ = 2869.92, d.f. = 66, *p* < .001). The Kaiser-Meyer-Olkin is a measure of sampling adequacy used to examine the appropriateness of factor analysis, whereas the test of sphericity has as a null hypothesis that the correlation matrix is an identity matrix and thus the factor model is inappropriate.

The results in Table 2 suggest a clear and simple structure for the 12 items of the SDQ-12, which perfectly match the theoretically relevant proposed dimensions of mystification and manipulation. Alpha coefficients for each factor were.81 and.81, respectively. The alpha coefficient for the complete scale was.85, indicating an adequate internal consistency.

**Table 2:** Items, factor loadings, means and standard deviations of mystification and manipulation scales (N = 541) ^1^.

Item	Factor 1 Mystification	Factor 2 Manipulation	Drug Abusers Mean (S.D.)	Alcohol Abusers Mean (S.D.)	General Population Mean (S.D.)
I do not seem to learn from certain mistakes I make in my life	.82		3.72 (1.26)	3.63 (1.26)	2.76 (1.14)
In important personal matters in my life, I think I repeatedly make the same mistakes	.76		3.65 (1.20)	3.61 (1.11)	2.59 (1.10)
I often recognize that others see my problems before (and better than) I do	.75		3.28 (1.24)	3.57 (1.16)	2.17 (0.99)
It takes me a while to become aware of certain key issues in my life	.75		3.30 (1.26)	3.18 (1.26)	2.21 (1.01)
It has been suggested (even if not specifically told) to me that I am wrong about my life	.72		3.54 (1.10)	3.52 (1.22)	2.02 (0.82)
I sometimes feel my lifestyle is a sham. I live a lie	.70		3.01 (1.38)	2.90 (1.33)	1.79 (0.90)
I resort to emotional blackmailing when needed		.82	2.98 (1.32)	2.50 (1.25)	2.18 (1.05)
I never resort to emotional manipulation*		-.82	2.78 (1.27)	3.23 (1.29)	3.40 (1.14)
I have been told (or it has been insinuated to me) that I manipulate people		.81	3.19 (1.45)	2.51 (1.34)	1.91 (1.05)
Honestly, I am one for changing things for my own convenience		.60	3.37 (1.21)	2.54 (1.11)	2.17 (0.96)
If you know me, you’ll say that I tend to lie and deceive in order to achieve my goals		.57	2.87 (1.34)	2.33 (1.24)	1.69 (0.88)
Honestly, I often choose to answer not with the truth but rather with whatever is most convenient for me		.53	2.73 (1.21)	2.54 (1.11)	2.17 (0.96)
Mean (S.D.)	18.78 (5.56)	16.28 (5.50)			

^1^ Factor loadings lower than.35 not shown

*Reversed score

### Confirmatory factor analyses

We first tested a single-factor model in which all 12 items of the SDQ-12 were indicators of a unique latent variable—self-deception–. This model fit the data very poorly (S-B χ^2^ = 462.83, d.f. = 54, p < .001, CFI = .84, RMSEA = .10, 90% C.I., .09, .11), suggesting that a single-factor solution was not adequate to explain the sample data and that the SDQ-12 cannot be described as unidimensional. Next, we used the dimensions theoretically proposed and empirically obtained through exploratory factor analysis to test a two-factor model. This model was equivalent to the model tested in the exploratory factor analysis. Mardia’s normalized estimate was 21.05, indicating a significant departure from multinormality and suggesting the use of the Satorra-Bentler χ^2^ to estimate fit indexes and standard errors. The results of model 1 indicated that estimating the correlation between errors associated with two items (*In important personal matters in my life*, *I think I repeatedly make the same mistakes* and *I do not seem to learn from certain mistakes I make in my life*) would produce a substantial reduction in χ^2^ (ΔS-B χ^2^ = 62,5, d.f. = 1, p < .001). The covariance between residuals is interpreted as an amount of variance not relevant to the construct and might have several causes (for example, item wording or format). This situation was correctly identified by the model.

This model showed adequate fit (S-B χ^2^ = 138.94, d.f. = 52, p < .001, CFI = .97, RMSEA = .05, 90% C.I., .03, .05). All items loaded statistically significantly on their corresponding factors (p < .001) in the final model. The correlation between the factors was statistically significant (r = .70, p < .001).

Finally, we tested the relationship of the factors with sex and age. To do so, we modeled both sex and age as exogenous variables predicting factor 1 and factor 2 scores. We also allowed covariates to freely covary. This model fit the data well (S-B χ^2^ = 175.94, d.f. = 72, p < .001, CFI = .96, RMSEA = .05, 90% C.I., .04, .05). The scores for the manipulation factor were significantly influenced by sex (β = -.25, p < .001) and age (β = -.12, p < .01), suggesting that higher scores on this factor were associated with male and younger respondents. Mystification showed no statistical relationship with age (β = .06, ns) or sex (β = 0.04, ns).

### Using the mystification and manipulation scores to differentiate between groups

To further check the validity of the mystification and manipulation scale scores, separate multivariate analyses of variance (MANOVA) were performed. These MANOVA were conducted to ascertain whether the means for mystification and manipulation were the same across groups of different populations. To do so, we compared the mean scores from the general population group with the mean scores for the two other groups: alcoholics and drug abusers. Given the differing compositions of the groups (the general population, the alcoholics and the drug abusers) in terms of sex and age, we controlled for the spurious influence of sex and age in the models tested. The effects of the interactions of group X sex and group X age were estimated. If these interactions were nonsignificant, we could conclude that main (group) effects were of interest. Nonsignificant interaction effects were found for the two groups: alcoholics (sex X alcohol—F2, 272 = 1.01, p = .37-; age X alcohol—F4, 546 = 1.01, p = .57) and drug abusers (sex X drug abuse—F2, 374 = .92, p = .40-; age X drug abuse—F4, 750 = 1.30, p = .27).

The main effects for group were all statistically significant (alcohol—F2, 272 = 47.52, p < .001-; drug abuse—F2, 374 = 55.86, p < .001). These results indicated that statistically significant main effects were independent of group composition in terms of sex and age. Univariate analyses of variance (ANOVA) showed that the means for mystification and manipulation were significantly different between the general population and the two target groups. For mystification, the general population group scored significantly lower (M = 13.54, S.D. = 3.65) than alcoholics (M = 20.39, S.D. = 5.25) (F1, 383 = 175.34, p < .001) and drug abusers (M = 20.49, S.D. = 5.17) (F1,280 = 152.53, p < .001). The same applied to manipulation, with scores for the general population group (M = 12.95, S.D. = 3.68) significantly lower than those for alcoholics (M = 15.68, S.D. = 5.32) (F1, 383 = 94.96, p < .001) and drug abusers (M = 18.36, S.D. = 5.59) (F1,280 = 23.14, p < .001).

### Discussion

In the present investigation, a new instrument of self-deception (SDQ-12) was developed by simplifying a previous, more extensive questionnaire (IAM-40). The SDQ-12 is not a short version of the original questionnaire. Rather, it restructures the IAM-40 in order to briefly provide valid and reliable information on two essential aspects of self-deception: manipulation and mystification. With this objective, the SDQ-12 was developed and validated based on responses from 541 subjects, which included clinical samples (alcoholics and drug addicts) and the general adult population.

The results suggest that the SDQ-12 allows precise detection of self-deception in two important components: manipulation and mystification. The manipulation subscale is very similar to the original subscale of the IAM-40; however, it is shorter (two fewer items) and refers to respondents’ self-presentation with the intention of influencing others’ behavior. The subscale of mystification measures respondents’ departure from reality that affects subjects’ vital activity and typically ends up in the creation of a shield of distrust that hinders interpersonal communication with communicative opacity and the systematic use of denial [22].

As it is an abbreviated and simpler questionnaire to administer, it reduces the fatigue, frustration and boredom that having to respond to similar questions might cause in the target population, making them less willing to be evaluated [23, 24].

The mystification subscale is the newest contribution of the SDQ-12, and it can be used as the test of choice (screening) to apply to groups such as addicts [2, 9] and subjects with personality disorders (for example, to determine self-deception in a population of inmates) [25, 39].

Studies on relapse prevention in addicts [26] show an inverse relationship between self-deception and the duration of abstinence: the longer the abstinence period lasts, the more likely that the relapse was caused by factors related to self-deception [26, 27]. Aspects such as overconfidence and wanting to prove or consider oneself already cured, are typical conditions of clinical self-deception in addicts.

Although our results do not allow for a direct comparison of the IAM-40 and the SDQ-12, the IAM-40 allows one to evaluate the progress of subjects in the five factors of self-deception that it explores (for example, in specific treatment centers that monitor therapeutic advances), whereas the SDQ-12, in addition to having the advantage of simplicity and rapidity of application, serves to quantify and qualify the level of pathological self-deception of respondents [25].

In summary, our study supports the clinical and research importance of the SDQ-12 for evaluating self-deception. In this sense, a large number of the available instruments focus on the measurement of related aspects, such as desirability and the management of impressions. The SDQ-12 is especially appropriate for evaluating the substantial components of pathological self-deception, and it can be applied not only in the addicted population [28, 29] but in multiple populations where mental health is susceptible to recurrence, especially those that follow an episodic course. Pathological self-deception is important in addictive and personality disorders. However, it is also relevant in other psychopathological processes, such as factitious disorders [30] and alimentary disorders [31] (for example, an anorexic ignores their fear of public rejection). It has even come under question in terms of its role in certain delusional disorders [32], and in induced pretense or simulation [32]. Alternatively, there are also studies [33] that show the role of self-deception in positive psychological aspects, whereby decreasing self-deception may improve these aspects (for example, self-esteem). In a book compiled by Myslobodskyi [34], different authors examined diverse forms of deception and self-deception in the context of different syndromes and neuropsychiatric diseases, concluding that falsehood in perception, cognition, and behaviors was a common thread.

This study also offers limitations, such as those related to internal validity. Future investigations should control for the effect of personality, contextual variables and the differential effect of various types of substance abuse that could distinctly affect the results. In addition, external validity must be ensured through control in the experimental assignment of groups, avoiding possible selection biases. Likewise, research on certain adaptive and neurotic disorders in general is required.

The levels of self-deception detected by the SDQ-12 can also be used in psychotherapy. For example, they can be an important aid in models such as the transtheoretical integrative model [35–38], based on an Ishikawa diagram to dismantle self-deception from a lower to higher intensity gradually, progressively [39] and absolutely uncritically to neutralize the habitual defensiveness of the addict.

The SDQ-12 might aid in the determination of the subject’s degree of mystification and tendency to manipulate. Given the limited trajectory of the questionnaire, the data are still incipient although encouraging in terms of the detection and control of self-deception in general and of mystification in particular. In its current state of development, the SDQ-12 has not yet been shown to be sensitive to changes, so additional research is needed to verify if its use is adequate for interventions. The results of these and subsequent investigations can guide therapists in their diagnostic work and psychotherapeutic interventions based on the proposed self-deception exploration model and subsequent suggestions from the line of intervention. This conclusion is hypothetical, and future research should verify it.
